# Ethical Concerns of AI in Neurosurgery: A Systematic Review

**DOI:** 10.1002/brb3.70333

**Published:** 2025-02-11

**Authors:** Muhammad Mohsin khan, Gianluca Scalia, Noman Shah, Giuseppe Emmanuele Umana, Vishal Chavda, Bipin Chaurasia

**Affiliations:** ^1^ Department of Neurosurgery Hamad General Hospital Doha Qatar; ^2^ Department of Clinical Research Dresden international university Dresden Germany; ^3^ Neurosurgery Unit, Department of Nead and Neck Surgery Garibaldi Hospital Catania Italy; ^4^ Department of Neurosurgery Trauma and Gamma Knife Center, Cannizzaro Hospital Catania Italy; ^5^ Department of Medicine Multispeciality, Trauma and ICCU Centre, Sardar Hospital Ahmedabad Gujarat India; ^6^ Department of Neurosurgery Neurosurgery Clinic Birgunj Nepal

**Keywords:** Artificial Intelligence, ethical considerations, ethics, neurosurgery, regulatory oversight, responsible AI, systematic review, transparent decision‐making

## Abstract

**Background:**

The relentless integration of Artificial Intelligence (AI) into neurosurgery necessitates a meticulous exploration of the associated ethical concerns. This systematic review focuses on synthesizing empirical studies, reviews, and opinion pieces from the past decade, offering a nuanced understanding of the evolving intersection between AI and neurosurgical ethics.

**Materials and Methods:**

Following PRISMA guidelines, a systematic review was conducted to identify studies addressing AI in neurosurgery, emphasizing ethical dimensions. The search strategy employed keywords related to AI, neurosurgery, and ethics. Inclusion criteria encompassed empirical studies, reviews, and ethical analyses published in the last decade, with English language restriction. Quality assessment using Joanna Briggs Institute tools ensured methodological rigor.

**Results:**

Eight key studies were identified, each contributing unique insights to the ethical considerations associated with AI in neurosurgery. Findings highlighted limitations of AI technologies, challenges in data bias, transparency, and legal responsibilities. The studies emphasized the need for responsible AI systems, regulatory oversight, and transparent decision‐making in neurosurgical practices.

**Conclusions:**

The synthesis of findings underscores the complexity of ethical considerations in the integration of AI in neurosurgery. Transparent and responsible AI use, regulatory oversight, and mitigation of biases emerged as recurring themes. The review calls for the establishment of comprehensive ethical guidelines to ensure safe and equitable AI integration into neurosurgical practices. Ongoing research, educational initiatives, and a culture of responsible innovation are crucial for navigating the evolving landscape of AI‐driven advancements in neurosurgery.

## Graphical abstract

The integration of Artificial Intelligence (AI) into neurosurgery necessitates a meticulous exploration of the associated ethical concerns. This systematic review and PRISMA charts suggests 8 key studies among 79 were identified to be associated with ethical concern related with AI in neurosurgery. It is imperative that ethical consideration should keep pace to ensure the safe, fair, and equitable integration of AI into neurosurgical practices.

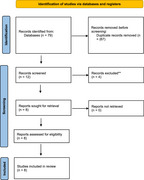

## 1 Introduction

The relentless advancement of Artificial Intelligence (AI) has propelled its widespread integration across diverse medical domains, and neurosurgery stands at the forefront of this transformative wave. This systematic review embarks on a meticulous examination and synthesis of empirical studies, comprehensive reviews, insightful opinion pieces, and ethical analyses that have unfolded within the realm of AI in neurosurgery over the past decade. By concentrating on the ethical dimensions inherent in the marriage of AI and neurosurgical practices, this review aims to provide a nuanced understanding of the ethical considerations accompanying the rapid evolution of technology in this specialized field of medicine. As AI technologies continue to permeate the fabric of healthcare, it becomes increasingly imperative to subject their integration into neurosurgery to rigorous scrutiny. This scrutiny is not merely to delineate the technological capabilities and clinical advancements but also to delve into the ethical implications that arise with the assimilation of AI in the intricate landscape of neurosurgical procedures. The objective is to navigate the ethical landscape surrounding AI in neurosurgery, offering insights that contribute to the formulation of responsible and safe implementation strategies. With an emphasis on the past decade, this review seeks to encapsulate the latest developments and prevailing ethical discourse, shedding light on the multifaceted intersection of artificial intelligence and neurosurgical ethics. Supporting information

## 2 Materials and Methods

**FIGURE 1:**
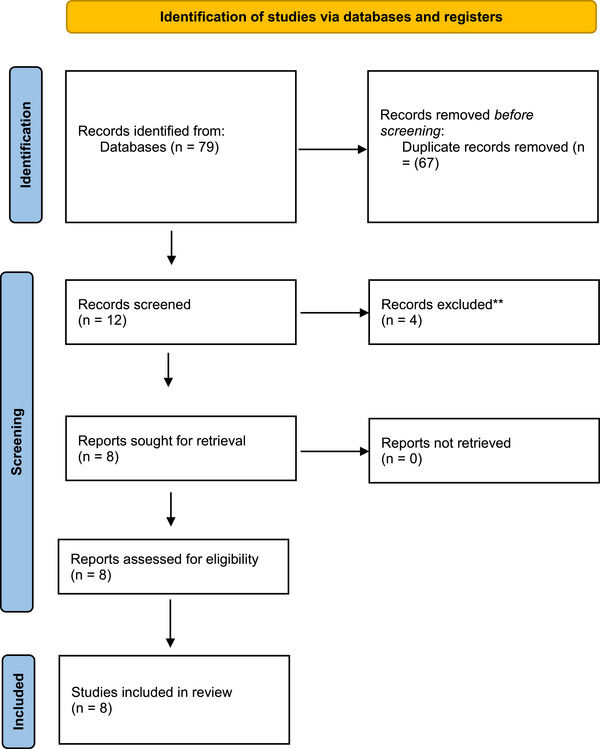
PRISMA flow chart.

The systematic review was conducted following the Preferred Reporting Items for Systematic Reviews and Meta‐Analyses (PRISMA) guidelines (**Figure** **1**). A comprehensive literature search was conducted across multiple databases including PubMed, IEEE Xplore, Scopus, and Google Scholar. The search strategy aimed to identify relevant studies using the following keywords: (“artificial intelligence” OR AI OR “machine learning” OR “neural networks” OR “computational intelligence”) AND (neurosurgery OR “neurosurgical procedures” OR “brain surgery” OR “spinal surgery” OR neurosurgical) AND (ethics OR “ethical concerns” OR “ethical issues” OR “moral implications”). Inclusion criteria encompassed empirical studies, reviews, opinion pieces, and ethical analyses specifically addressing AI in neurosurgery. To ensure the currency of information, only studies published within the last decade were included. The language of publication was limited to English, and the selected studies had to be available in full‐text form. Exclusion criteria involved studies not directly related to AI in neurosurgery or those lacking a focus on ethical concerns. Quality assessment was conducted using critical appraisal tools provided by the Joanna Briggs Institute. Two independent reviewers assessed each study for methodological rigor, credibility of sources, coherence of arguments, and relevance to the research questions. This rigorous quality assessment informed the subsequent analysis and synthesis of the evidence, contributing to the overall reliability and validity of the review findings.

## 3 Results

The systematic review identified seven key studies addressing ethical concerns associated with AI in neurosurgery. Each study provided unique insights into the ethical implications of incorporating AI technologies in neurosurgical practices (**Table** **1**).

**TABLE 1:** Literatures showing ethical implications of incorporating AI technologies in neurosurgical practices.

Study	Title	Journal/source	Year	Main objective	Methodology	Key findings	Ethical considerations
**Kuang et al**.	ChatGPT Encounters Multiple Opportunities and Challenges in Neurosurgery	*International Journal of Surgery*	2023	Investigate the potential of ChatGPT in neurosurgery	Inquiry and analysis	ChatGPT has limitations in depth, personalization, and clinical application	Legal and ethical concerns in healthcare AI, need for human oversight
**Naik et al**.	Legal and Ethical Consideration in Artificial Intelligence in Healthcare	*Frontiers in Surgery*	2022	Address legal and ethical issues in AI in healthcare	Review	Challenges in data bias, transparency, fairness, informed consent, and legal responsibilities	Need for responsible AI systems, transparent and ethical decision‐making
**Noh et al**.	Artificial Intelligence for Neurosurgery: Current State and Future Directions	*Journal of Korean Neurosurgical Society*	2023	Understand AI's role in neurosurgery and its future development directions	Review	AI's role in diagnostic imaging, robotic neurosurgery, neurointensive care, and prognosis prediction	Emphasis on understanding AI for enhancing patient care in neurosurgery
**Ray et al**.	The Perils and Promises of Generative Artificial Intelligence in Neurointerventional Surgery	*Journal of NeuroInterventional Surgery*	2023	Explore benefits and risks of generative AI in neurointerventional surgery	Commentary	Potential of AI for surgical precision and diagnosis accuracy; challenges include data biases and ethical issues	Need for careful regulation and oversight
**Roman et al**.	The Expanding Role of ChatGPT in Neurosurgery: A Systematic Review	*Cureus*	2023	Explore the use of ChatGPT in neurosurgery	Systematic literature review	ChatGPT's potential in surgical planning, diagnosis, and patient care; limitations in data requirements and output errors	Need for responsible AI use and human oversight
**Green**	Ethical Reflections on Artificial Intelligence	*SetF, 6(2)/2018*	2018	Explore the ethical implications of AI in various fields, including neurosurgery.	Philosophical and ethical analysis	AI impacts positive and negative; ethical concerns include safety, bias in data, AI‐induced unemployment, etc.	Safety and reliability of AI systems, bias in AI due to training data, economic and social implications of AI
**Yirmibesoglu Erkal et al**.	Ethical Evaluation of Artificial Intelligence Applications in Radiotherapy Using the Four Topics Approach	*Artificial Intelligence In Medicine, 115 (2021) 102055*	2021	Address the ethical concerns in the use of AI in radiotherapy, applicable to neurosurgery.	Review and analysis	AI's role in clinical decision‐making raises ethical concerns	Maintaining patient‐physician relationship integrity, addressing biases in AI
Bečulić et al.	ChatGPT's Contributions to the Evolution of Neurosurgical Practice and Education: A Systematic Review of Benefits, Concerns and Limitations	*Medicinski Glasnik*	2024	To review the potential benefits and limitations of ChatGPT in neurosurgical practice and education	Systematic review using PRISMA guidelines; 13 studies analyzed	ChatGPT shows potential to enhance neurosurgical practice, including personalized treatment plans, surgical support, and data processing. Risks include validation challenges, algorithmic bias, and question format limitations	Highlights the importance of validation for accuracy and addressing ethical concerns like data security and algorithmic bias

Kuang et al. (2023) highlighted the limitations of ChatGPT in depth, personalization, and clinical application, emphasizing the necessity of legal and ethical considerations in healthcare AI. This underscores the importance of human oversight in AI applications in neurosurgery.

Naik et al. (2022) conducted a comprehensive review, addressing challenges such as data bias, transparency, fairness, informed consent, and legal responsibilities in AI in healthcare. Their findings underscored the critical need for responsible and transparent AI systems in neurosurgical practices.

Noh et al. (2023) provided a forward‐looking perspective on AI's role in neurosurgery, emphasizing its potential in diagnostic imaging, robotic neurosurgery, neurointensive care, and prognosis prediction. The study encourages a proactive approach in understanding and integrating AI for enhanced patient care.

Ray et al. (2023) explored the perils and promises of generative AI in neurointerventional surgery, acknowledging its potential for precision and accuracy while cautioning against data biases and ethical issues. The study highlights the importance of regulatory oversight in the integration of AI in neurosurgical procedures.

Roman, Al‐Sharif, and Al Gharyani (2023) conducted a systematic review of ChatGPT's expanding role in neurosurgery, emphasizing its potential in surgical planning and diagnosis. The study underscores the need for responsible AI use, considering the limitations in data requirements and potential output errors.

Green (2018) provided a philosophical and ethical analysis of AI, addressing safety, reliability, biases, and the economic and social implications of AI. This study emphasizes the overarching ethical concerns that extend beyond individual applications, providing a broader perspective.

Yirmibesoglu Erkal, Akpınar, and Erkal (2021) focused on ethical concerns in AI's role in clinical decision‐making in radiotherapy, applicable to neurosurgery. The study stresses the importance of maintaining the integrity of the patient‐physician relationship and addressing biases in AI for ethical AI implementation.

Bečulić et al. (2024) highlighted ChatGPT's potential to revolutionize neurosurgery by enhancing surgical planning, enabling personalized treatments, and improving data processing efficiency. However, they also underscored significant challenges, including issues with output validation, algorithmic bias, and ethical concerns such as data security and the risks of overreliance. Their study emphasizes the need for rigorous validation and ethical oversight to ensure the responsible integration of generative AI into clinical practice.

In conclusion, the collective findings from these studies underscore the complexity of ethical considerations in the integration of AI in neurosurgery. Transparency, bias mitigation, regulatory oversight, and responsible AI use emerge as recurrent themes, emphasizing the need for a holistic and ethical approach in the adoption of AI technologies within neurosurgical practices. These considerations contribute to shaping the ongoing discourse on ethical AI development and deployment.

## 4 Discussion

The integration of Artificial Intelligence (AI) into neurosurgery has introduced a complex array of ethical considerations. This synthesis aims to delve into the key findings of selected studies, shedding light on recurrent themes that demand careful consideration and thoughtful discourse.

### 4.1 Understanding AI Limitations

One prominent theme revolves around the limitations and challenges associated with AI technologies. Notably, Kuang et al. (2023) highlight ChatGPT's shortcomings in depth, personalization, and clinical application. This underscores the importance of a nuanced understanding of AI capabilities in neurosurgery, acknowledging both its potential and limitations.

### 4.2 Addressing Pervasive Challenges in AI

Naik et al. (2022) bring attention to pervasive challenges in AI, encompassing data bias, transparency, fairness, informed consent, and legal responsibilities. The emphasis here is on the necessity of developing responsible and transparent AI systems in neurosurgical practices to maintain trust among various stakeholders.

### 4.3 Forward‐Looking Perspective on AI in Neurosurgery

Noh et al. (2023) provide a forward‐looking perspective on AI's role in neurosurgery, emphasizing its potential across various domains. From diagnostic imaging to robotic neurosurgery, neurointensive care, and prognosis prediction, the study encourages a proactive approach in understanding and integrating AI for enhanced patient care, with a concurrent emphasis on ethical considerations.

### 4.4 Promises and Perils of Generative AI

Ray et al. (2023) delves into the promises and perils of generative AI in neurointerventional surgery. While recognizing the potential for precision and accuracy, the study issues a cautionary note on data biases and ethical concerns. This dual perspective underscores the necessity for regulatory oversight and ethical guidelines in ensuring the safe and ethical deployment of generative AI in neurosurgical procedures.

### 4.5 ChatGPT's Role and Responsible AI Use

Roman, Al‐Sharif, and Al Gharyani (2023) focus on the expanding role of ChatGPT in neurosurgery, emphasizing its potential in surgical planning and diagnosis. Simultaneously, the study addresses limitations in data requirements and potential output errors, emphasizing the need for responsible AI use. Human oversight and a cautious approach are deemed crucial in mitigating ethical risks associated with AI applications.

### 4.6 Philosophical and Ethical Reflections on AI

Green (2018) contribute a broader perspective by offering philosophical and ethical reflections on AI across various fields, including neurosurgery. The study underscores overarching ethical concerns such as safety, reliability, biases, and socioeconomic implications. These reflections emphasize the importance of considering the broader societal impact of AI technologies beyond individual applications.

### 4.7 AI in Clinical Decision‐Making

Yirmibesoglu Erkal, Akpınar, and Erkal (2021) specifically focus on AI's role in clinical decision‐making in radiotherapy, applicable to neurosurgery. The study underscores the importance of maintaining the integrity of the patient‐physician relationship and addressing biases in AI for ethical AI implementation within the neurosurgical context.

### 4.8 Balancing Innovation and Ethics in AI Integration

Bečulić et al. (2024) critically analyze ChatGPT's role in neurosurgical practice, highlighting both its potential and limitations. While it shows promise in enhancing surgical planning, treatment personalization, and data analysis, the authors emphasize risks such as algorithmic bias, overreliance on AI, and threats to data privacy. They argue that unchecked reliance on AI could undermine human judgment and erode trust in clinical decision‐making. The study underscores the need for rigorous validation and ethical frameworks to ensure AI tools complement human expertise and prioritize patient welfare, calling for a balanced approach to integration.

The synthesis of these studies underscores the intricate web of ethical considerations entwined with the integration of AI in neurosurgery. Transparency, bias mitigation, regulatory oversight, and responsible AI use emerge as recurrent themes, emphasizing the imperative for a holistic and ethical approach in the adoption of AI technologies within neurosurgical practices. These considerations contribute significantly to the ongoing discourse on ethical AI development and deployment.

### 4.9 Implications and Future Research

Given the diverse ethical considerations discussed, there is a clear call for ongoing research and collaboration to refine and expand ethical frameworks. Educational initiatives for healthcare professionals, researchers, and the broader public can contribute to a shared understanding of AI's ethical implications in neurosurgery. Fostering a culture of responsible innovation and ethical practice is deemed essential as the field of AI in neurosurgery continues to evolve (Dagi, Barker, and Glass 2021; Dundar et al. 2022; El‐Hajj et al. 2023; Goswami and Kumar 2021; Kazemzadeh et al. 2023; Lim 2021; Mishra et al. 2022a,b; Mishra and Deora 2023; Mofatteh 2021; Palmisciano et al. 2020; Panesar et al. 2020; Ryvlin et al. 2023; Senders et al. 2018; Sevgi et al. 2023) Kingsly et al. 2023.

### 4.10 Limitations

This study has certain limitations that should be considered. The diverse methodologies, outcomes, and contexts of the included studies may limit the general applicability of the findings by focusing specifically on ChatGPT's applications in neurosurgery. The study may overlook broader implications or uses of this technology in other medical disciplines. Another key limitation is the rapidly advancing nature of AI technologies, which may outpace the relevance of some conclusions drawn from the current literature. Addressing these limitations in future research will be crucial to gaining a deeper and more comprehensive understanding of AI's role in clinical practice.

## 5 Conclusions

In conclusion, this systematic review underscores the critical need for the establishment of thorough and comprehensive ethical guidelines governing the use of AI in neurosurgery. As technology continues to advance, it is imperative that ethical considerations keep pace to ensure the safe, fair, and equitable integration of AI into neurosurgical practices. The development of robust ethical frameworks will not only safeguard patient well‐being but also foster trust and confidence in the evolving landscape of AI‐driven advancements in neurosurgery.

## Author Contributions

**Muhammad Mohsin khan**: Conceptualization, investigation, writing—original draft, formal analysis, software, data curation. **Gianluca Scalia**: Writing—review & editing, visualization, validation, methodology. **Noman Shah**: Validation, formal analysis. **Giuseppe Emmanuele Umana**: Investigation, validation, formal analysis. **Vishal Chavda**: Formal analysis, software, supervision. **Bipin Chaurasia**: Conceptualization, writing—review & editing, visualization, validation, supervision.

## Ethics Statement

To ensure transparency and adherence to ethical research practices, the study has been registered in the Open Science Framework (OSF). The full protocol is publicly accessible at DOI: 10.17605/OSF.IO/JP4CT.

## Conflicts of Interest

The authors declare no competing interests related to this review.

### Peer Review

The peer review history for this article is available at https://publons.com/publon/10.1002/brb3.70333.

## Supporting information



Supporting Information

## Data Availability

Data sharing not applicable to this article as no datasets were generated or analyzed during the current study.
